# A Review of 1073 Cases of Wide-Awake-Local-Anaesthesia-No-Tourniquet (WALANT) in Finger and Hand Surgeries in an Urban Hospital in Malaysia

**DOI:** 10.7759/cureus.16269

**Published:** 2021-07-08

**Authors:** Shalimar Abdullah, Lim Chia Hua, Lau Sheau Yun, Alexander Samuel Thavamany Devapitchai, Amir Adham Ahmad, Parminder Singh Gill Narin Singh, Jamari Sapuan

**Affiliations:** 1 Hand and Microsurgery Unit, Department of Orthopaedics and Traumatology, Faculty of Medicine, Universiti Kebangsaan Malaysia Medical Centre, Kuala Lumpur, MYS; 2 Department of Orthopaedics and Traumatology, Faculty of Medicine, Universiti Kebangsaan Malaysia Medical Centre, Kuala Lumpur, MYS; 3 Faculty of Medicine, Universiti Kebangsaan Malaysia Medical Centre, Kuala Lumpur, MYS; 4 Hand and Microsurgery Unit, Department of Orthopaedics and Spine Surgery, Prince Court Medical Centre, Kuala Lumpur, MYS

**Keywords:** lignocaine, lidocaine, epinephrine, adrenaline, digital ischaemia

## Abstract

Background

The Wide-Awake-Local-Anaesthesia-No-Tourniquet (WALANT) technique achieves an almost bloodless field for clear visualization during surgeries. WALANT utilizes lidocaine and epinephrine for anesthesia and hemostasis, respectively, without the usage of sedation and tourniquet. This avoids the potential side effects of tourniquet-related pain and sedation-related complications. However, acceptance is still low due to concerns regarding the safety of epinephrine injection in the finger. There is a persistent belief that epinephrine can cause digital ischemia.

Purpose

To evaluate retrospectively possible complications of hand surgeries performed using the WALANT technique.

Methods

All finger and hand procedures performed under the WALANT technique from June 2016 to May 2021 in an urban tertiary hospital were studied retrospectively.

Results

There were a total of 1073 cases, of which 694 were females and 379 were males. The mean age was 55 years. Finger surgeries (e.g., trigger finger release, excision of finger lesions, removal of implants) consisted of 707 cases; and the rest (366 cases) were hand surgeries (e.g., carpal tunnel release, excision of hand lesions, removal of implants). In all cases reviewed, there were no instances of circulatory compromise. There were also no circumstances where usage of reversal with phentolamine is recorded.

Conclusion

We believe that performing finger and hand surgeries using the WALANT technique is safe and beneficial. The usage of WALANT in hand surgeries avoids tourniquet pain. However, WALANT should be used with caution in those with vascular insufficiency or disease.

## Introduction

The Wide-Awake-Local-Anaesthesia-No-Tourniquet (WALANT) technique is performed purely under local anesthesia and has gained popularity among hand surgeons, as it offers a wide range of advantages to both surgeons and patients. In this approach, epinephrine is used as an alternative to a tourniquet to provide an almost bloodless field for better visualization during hand surgeries. Tourniquet usage is known to cause discomfort and pain in patients and may require sedation. The WALANT technique avoids tourniquet-related pain and possible sedation-related complications [1].

Many still believe that epinephrine can cause finger necrosis despite publications of many articles on WALANT usage [2-5]. We intend to review a large number of patients to investigate the occurrence of digital ischemia in selected patients undergoing WALANT.

## Materials and methods

After obtaining approval from our institutional review board, all the medical records of hand surgeries performed using the WALANT technique from June 2016 to May 2021 were reviewed retrospectively. All surgeries were done at Universiti Kebangsaan Malaysia Medical Center, Kuala Lumpur, Malaysia. The diagnosis, type of procedure, and any instances of complication or usage of reversal were reviewed in the medical records. Usage of reversal is defined as any incidence where phentolamine is required.

All surgeries were done in the minor operating room in a day-care setting. We utilized a mixture of lignocaine 1% with adrenaline 1:100,000 and 1 ml of 8.4% sodium bicarbonate. The injection was administered a minimum of 30 minutes prior to the procedure in the treatment room of the day-care surgery complex. Skin preparation was done with an alcohol swab. A 26-gauge needle was injected 90 degrees perpendicularly to the skin, just over the most proximal area of the surgery, and depending on the area of the surgery, 4-8 ml of anesthesia was injected. Finger surgeries, such as trigger finger, releases required approximately 4-5 ml of the injection solution, whilst hand surgeries such as carpal tunnel releases required 7-8 ml of injection solution. In cases where more than one was finger injected, it was recorded as more than one case.

## Results

The mean age of patients reviewed was 55 years old (from 8-92 years old). This included 379 males and 694 females. The racial composition consisted of 696 Malays, 287 Chinese, 77 Indians, five Sikhs, and two from the indigenous population. The remaining six patients were from other nationalities, i.e., Myanmar, Indonesia, and Russia.

In all 1073 cases, there were no incidences of circulatory compromise observed. There were also no instances where reversal with phentolamine was required.

The most common types of finger procedures performed were trigger finger release (536 cases), followed by excision of the lesion (82 cases), and removal of implant (23 cases). Other procedures include wound debridement, k-wiring, and removal of the foreign body as shown in Table 1.

**Table 1:** Finger procedures that were done under WALANT WALANT: Wide-Awake Local Anaesthesia No Tourniquet

Procedure	Number of Cases							
	Age					Gender		
	<18	18-30	31-50	>50	Total	Male	Female	Total
Trigger finger release	2	3	78	453	536	175	361	536
Excision of finger lesion	4	10	30	38	82	32	50	82
Removal of implant	1	8	6	8	23	18	5	23
Wound debridement	0	1	4	3	8	5	3	8
K-wiring	2	3	4	2	11	10	1	11
Open reduction internal fixation	0	2	4	3	9	3	6	9
Foreign body removal	0	4	2	1	7	4	3	7
Ingrown nail procedure	0	0	2	2	4	2	2	4
Manipulation under anesthesia	1	1	1	0	3	1	2	3
Incision and drainage	0	1	1	2	4	2	2	4
Adhesiolysis	0	0	4	1	5	4	1	5
Extensor tendon repair	0	2	1	1	4	4	0	4
Release of skin flap/soft tissue/muscle/tendon or contracture	0	0	1	1	2	2	0	2
Amputation	0	1	1	1	3	3	0	3
Flexor tendon repair	0	0	2	0	2	1	1	2
Skin graft	0	0	1	0	1	0	1	1
Nail excision	0	0	1	1	2	0	2	2
Webbing of finger post-burn	0	0	0	1	1	0	1	1
Total	10	36	143	518	707	266	441	707

The most common type of hand procedure performed was carpal tunnel release (291 cases) followed by excision of the lesion (29 cases) and removal of implant (21 cases). Others are shown in Table 2.

**Table 2:** Hand procedures that were done under WALANT WALANT: Wide-Awake Local Anaesthesia No Tourniquet

Procedure	Number of Cases							
	<18	18-30	31-50	>50	Total	Male	Female	Total
Carpal tunnel release	0	9	105	177	291	65	226	291
Excision of hand lesion	1	7	13	8	29	11	18	29
Removal of implant	0	5	10	6	21	18	3	21
DeQuervain’s surgery	0	2	1	2	5	3	2	5
Open reduction internal fixation	0	8	3	0	11	9	2	11
Fixation of small bone	0	1	1	1	3	3	0	3
Foreign body removal	0	1	0	0	1	1	0	1
Manipulation under anesthesia	0	0	0	1	1	1	0	1
Wound exploration	0	0	0	1	1	1	0	1
Guyon canal release	0	0	0	2	2	0	2	2
Needle aponeurectomy	0	0	0	1	1	1	0	1
Total	1	33	133	199	366	113	253	366

All these procedures were done in the minor operation theater instead of the major operation room. This reflects a possibility of shifting many hand surgeries from major operation theater to minor operation theater or procedure room with field sterility.

## Discussion

Finger surgeries are commonly done without the usage of a tourniquet but hand surgery utilizes the tourniquet. Traditionally, the tourniquet was used to provide an almost bloodless field for better visualization during procedures involving the extremities. However, usage of the tourniquet is associated with discomfort and pain either at the site of the tourniquet or the limb distal to the cuff. Tourniquet pain was described as initial pressure sensation followed by progressing numbness and paralysis which later progressed to severe aching pain. Deflation of the cuff was associated with intense vibratory pain as well [6]. Thus, the WALANT technique that uses epinephrine as an alternative to achieve hemostasis is preferred by many hand surgeons.

It is commonly taught and written in many medical textbooks that epinephrine can cause finger necrosis and thus should not be injected into the finger. This myth was disproved when a critical review on cases of finger necrosis associated with local anesthesia and epinephrine injection from 1880 to 2000 was done [3]. There are several reasons to suggest that epinephrine should not be blamed.

First, out of the 48 cases of finger infarction, 27 cases were injected with local anesthesia alone without the involvement of epinephrine. It follows that local anesthesia should be the culprit for finger necrosis rather than epinephrine. In the rest of the 21 cases, epinephrine was injected along with local anesthesia. Second, a majority of the cases occurred before the 1950s when procaine was the only local anesthesia in usage. Procaine by itself was known to cause necrosis at that time. Acidic procaine with a pH as low as 1 was utilized. This acidic procaine is toxic and can cause finger infarction. Furthermore, there was no attempt to reverse these incidences using phentolamine, which was manufactured only after 1950.

An extensive review was done by Fitzcharles-Bowe et al. from the year 1900 to 2005 on accidental injection of high dose epinephrine (1:1000) in the finger of 59 cases reported no instances of digit necrosis or skin loss reported [7].

In a multicentre prospective study of 3110 cases of elective epinephrine injection in the hand and fingers by Lalonde et al., there were no instances of finger infarction, skin necrosis, or tissue loss of any kind. There were no incidences where reversal was required [8]. A review of 611 cases by Saeed et al. in 2010 also reported no complications with the use of epinephrine in digital blocks [9].

A recent article by Zaim et al. on 43 patients who received WALANT for trigger finger release reported no incidence of digital ischemia [10]. Gunasegaran et al. injected WALANT in 18 patients undergoing a variety of trigger finger, carpal tunnel, and ganglion surgeries without any adverse complications [11]. Thus, finger injection of low-dose epinephrine (1:100000) is believed to be safe.

In China, Tang et al. [12] have estimated hundreds of surgeries done under WALANT. In Nantong, China more than 2000 patients have undergone WALANT surgery in the past 6 years. In Tianjin, roughly 500 patients had WALANT surgery in one year. Tang et al. estimates about 100 Chinese hand surgeons and 10 hospitals regularly use WALANT [12]. There is no explicit mention of the epinephrine concentration but there had been no complications from the usage of epinephrine [12].

In the year 2018, Ayhan et al. demonstrated their experience of using the WALANT technique over a two-year duration [13]. Out of the 682 hand procedures done, there were no instances of finger loss. However, there was circulatory compromise observed in two fingers. In the first case, a higher amount of local anesthesia than planned was accidentally injected. The circulatory compromise resolved spontaneously without the usage of a reversal agent. In the second case of a middle phalanx fracture after a crush injury with circulatory compromise, the circulatory status prior to the operation was unknown. Phentolamine was successful in restoring circulation.

WALANT should be used with caution in fingers with possible circulatory compromise [14]. These include those with Buerger’s disease, Raynaud phenomenon, thrombotic disease, scleroderma, or after a severe crush injury. Previous incidences of digital ischemia have occurred in patients with the Raynaud phenomenon and those with diabetes mellitus and ischaemic cerebral infarct [15-16]. Zhu et al. reported a case of delayed onset of finger ischemia after injection with local anesthesia and epinephrine, which lasted for 14 hours until rescued with phentolamine [17]. Articles by Ruiter et al. and Zhang et al. reported cases of fingers necrosis that subsequently lead to finger amputation in digits injected with epinephrine [18-19]. In both studies, reversal with phentolamine was not attempted. We have summarized the known cases of digital ischemia in finger surgeries under WALANT in Table 3.

**Table 3:** A summary of articles reporting finger ischemia or circulatory compromise post-WALANT injection in finger LA: local anesthesia; WALANT: Wide-Awake Local Anaesthesia No Tourniquet

Article- Year and Journal	Authors	Case	Short history of the case	Predisposing factors	Duration before ischemia occurs post-injection	Management and final outcome
2018 Hand Microsurg	Ayhan E, Ozdemir E, Gumusoglu E, et al. [13]	1. Trigger finger release, 2. Middle phalanx fracture	Injection of a larger amount of LA (12 mls) than planned surgery for middle phalanx fracture	1. None identified; 2. Crush injury	1. 4 hours; 2. 2 hours	In the first patient, circulatory compromise resolved spontaneously. In the second patient, phentolamine rescue was injected.
2017 J Hand Surg Am	Zhu AF, Hood BR, Morris MS, et al. [17]	Carpal tunnel and right middle finger trigger finger releases	Developed late-onset finger ischemia LA (low-dose epinephrine, 1:100,000). Twice-recommended dose of LA volume injected.	History of cold intolerance, cold weather (3 degrees Celsius), coronary artery disease with 4 stents	3 hours	Treated with phentolamine. Finger ischemia lasted for 14 hours.
2017 J Hand Surg Am	Zhang JX, Gray J, Lalonde DH, et al. [19]	A 63-year-old female with a three-finger trigger release	Developed digital necrosis following surgery under 1% lidocaine and epinephrine (1:100,000).	Smokes 1 pack a day (40-pack-year)	Nearly 24 hours	Finger amputation of the tip of the index and middle fingers. Ring finger allowed to heal conservatively. Reversal with phentolamine was not attempted as the presentation was nearly 24 hours.
2015 Ned Tijdschr Geneeskd	Hutting K, Van Rappard JR, Prins A, et al. [16]	70-year-old female undergoing trigger finger release	Developed digital ischemia following release under LA (1% lidocaine-epinephrine (1: 100,000) solution).	Diabetes mellitus. Ischaemic cerebral infarct.	Few hours	Nadroparin had no improvement but nifedipine improved symptoms. Digital necrosis developed in the affected fingers several weeks later. Postoperatively, the X-ray showed calcifications suggestive of severe atherosclerosis.
2014 Eplasty	Ruiter T, Harter T, Miladore N, et al [18]	16-year-old female undergoing skin lesion excision	Developed digital ischemia after receiving an injection of lidocaine and epinephrine	None	8 hours	Developed digital necrosis despite 1 week of inpatient care. Distal phalangeal amputation was done. No reversal with phentolamine.
2012 J R Coll Physicians Edinburgh	Ravindran V, Rajendran S [15]	19-year-old with acute paronychia	Developed digital necrosis after incision and drainage under LA (lignocaine and epinephrine)	Primary Raynaud’s phenomenon	Immediately	Conservative (prescribed a week’s course of oral antibiotics). No reversal with phentolamine.

Various methods have been described to restore circulation in cases of finger ischaemic such as local infiltration of phentolamine, topical infiltration with terbutaline, and systemic and topical nitro-glycerine [20]. Phentolamine is most commonly used and appears to be the most effective method. Adrenaline injected fingers require approximately 85 minutes to return to a normal color after phentolamine injection compared to 320 minutes without phentolamine injection. It is also notable that capillary refill remains present in the nail bed at all times despite color changes after the injection. This shows that there is a decrease but not an absence of blood flow to the finger [21].

Technique without using tourniquet and sedation has many benefits include avoiding tourniquet pain and increasing patient’s comfort. Gunasegaran et al. had reported a statistically significant lower pain score in the WALANT group compared to the local anesthetic and tourniquet group [11]. Zaim et al. reported that the return of sensation was longer at 6.86 hours for the WALANT group versus 4.09 hours for the lignocaine-only group conferring added comfort and this was statistically significant [10]. Both Gunasegaran et al. and Zaim et al. had no significant difference in the operation time and blood loss.

The limitation of our study is that we did not record the number of patients with diabetes undergoing finger or hand surgery. We did not categorize diabetes as a severe type of peripheral vascular disease and therefore did not exclude diabetic patients from undergoing WALANT procedures. We will also be unaware of patients who developed delayed complications who may present to another center post-WALANT technique. However, our orthopedic fraternity is small in our country and will usually be addressed to us even if presented to other centers.

In our study, it is notable that all the 1073 cases were performed in the minor operation room instead of the main operating room. Shifting of hand surgeries to the minor operation room reduces the cost of operation and increases the efficiency and productivity of surgeons.

A detailed analysis of the cost and efficiency of performing carpal tunnel release done in Canada shows increased efficiency with reduced cost when the procedure is performed in the ambulatory setting [4]. This is evidenced by nine surgeries done under an ambulatory setting versus four surgeries in the main operation room (costing two times more) within three hours. The ambulatory setting costs less than that in the operation room (USD 36 per case under ambulatory setting versus USD 137 per case in operation room). This is a good alternative in any setting that has limited resources and budget.

Other than the technique of anesthesia, surgery is performed in a normal fashion. Therefore, there should be no difference in terms of the outcome of the operation. Studies show that all the patients who underwent surgeries using the WALANT technique have successful and painless surgery. There were no extra bleeding or complications reported [5,22].

## Conclusions

In conclusion, we believe that performing finger and hand surgeries using the WALANT technique is safe. Usage of WALANT in hand surgeries avoids tourniquet pain. However, WALANT should be used with caution in those with vascular insufficiency or disease.
